# Acupoint herbal patching for bronchitis: A protocol systematic review of randomized controlled trials

**DOI:** 10.1097/MD.0000000000016368

**Published:** 2019-07-19

**Authors:** Ji Hee Jun, Kyeong Han Kim, Eunhye Song, Lin Anga, Sunju Park

**Affiliations:** a Clinical Medicine Division, Korea Institute of Oriental Medicine; b Department of Preventive Medicine, College of Korean Medicine, Daejeon University, Daejeon; c Department of Preventive Medicine, College of Korean Medicine, Woosuk University, Jeonju; d Global Research and Development Cooperation Team, Korea Institute of Oriental Medicine; e Korean Convergence Medicine, University of Science and Technology, Daejeon, Republic of Korea.

**Keywords:** acupoint herbal patching, bronchitis, meta-analysis, systematic review

## Abstract

**Background:**

Acupoint herbal patching (AHP) is widely used for symptom management in patients with acute and chronic bronchitis. The purpose of this protocol review is to evaluate the safety and efficacy of AHP for the treatment of bronchitis.

**Methods and analysis:**

This protocol of systematic review will be conducted in accordance with the Preferred Reporting Items for Systematic Review and Meta-analysis Protocols (PRISMA-P). The databases searched will include PubMed, Embase, CENTRAL, Web of Science, 3 Korean medical databases (OASIS, Korea Med, and KMBASE), and the Chinese database China National Knowledge Infrastructure (CNKI). Randomized controlled trials (RCTs) or quasi-RCTs using AHP for bronchitis will be considered. The selection of the studies, data abstraction, and validations will be performed independently by 3 researchers.

**Conclusion:**

The conclusion of the review will provide evidence that AHP is an effective intervention in patients with bronchitis.

**Ethics and dissemination:**

As individuals were not involved, ethical approval is not required. Findings will be published in a peer-reviewed journal. This systematic review may inform the treatment of bronchitis patients in clinical practice.

**Registration:**

This systematic review has been registered with the International Prospective Register of Systematic Reviews (PROSPERO). The reference number is CRD42018110380.

## 1 Introduction

Bronchitis can be defined as a condition in which the mucosal lining of the bronchial tubes is infected, inflamed, and swollen.^[1]^ This condition may be either acute or chronic. Acute bronchitis is a common short-term illness that usually improves within a few days or may last for some weeks, but generally resolves without lasting complications. Contrary to that, chronic bronchitis is a more serious long-term illness that usually lasts for more than 3 and recurs for at least 2 consecutive years.^[2,3]^ People suffering from bronchitis often present with primary symptoms such as cough, production of thick discolored mucus, varying degrees of breathing difficulties, chest discomfort, and sometimes mild fever or chills.^[4–6]^ Recent studies have shown that the prevalence of bronchitis is increasing worldwide, and the condition is gradually becoming one of the major public health concerns, regardless of age and sex.^[7–15]^

Acupoint herbal patching (AHP) is a treatment that prevents diseases by strengthening the immune system through the stimulation of acupuncture points with small quantities of various herbal preparations.^[16–18]^ It is a complex therapy as it uses both herbal medicine and acupoints. Herbal patches are applied to acupoints such as BL-13, BL-15, and BL-17 for 4 to 6 hours. The earliest record of AHP goes back to the classic “Prescriptions for Fifty-two Diseases” (Wushi’er Bingfang), where it was listed as a treatment method, and is still widely used today.^[19,20]^ It is a treatment method that prevents diseases that commonly occur in winter by replenishing the energy of summer. AHP is noninvasive, safe, inexpensive, and easy to use, which makes it suitable for children, adolescents, and the elderly.^[21,22]^ The number of patients using AHP has been steadily increasing over recent years in China and Korea.^[23]^ Statistics from China suggest that approximately 100,000 people are treated with AHP every summer, and the number has increased by 13.14% from 2013 to 2014. The Chinese Acupuncture Society and the China Academy of Chinese Medical Sciences have selected more than 10 hospitals in China where research on the clinical efficacy and safety of AHP is in progress.^[24]^

Animal studies suggest that AHP reduces immunoglobulin E and interleukin 4 serum levels while it increases interferon gamma serum levels and thus supports the treatment of respiratory diseases. In addition, the treatment can improve symptoms associated with pulmonary function disorders by increasing the forced expiratory volume in the 1st, 2nd, and peak expiratory flow.^[19]^

A report published by the Organization for Economic Cooperation and Development states that the 3 million premature of patients suffering from respiratory diseases due to fine dust is increasing worldwide.^[25]^ Various studies are being conducted to develop effective therapies for bronchitis, and the interest in herbal treatments has continuously grown. Recently, AHP has been reported to be widely used as an effective alternative treatment for respiratory diseases such as asthma and rhinitis.^[20,26,27]^ Most of the articles analyzed selected acupoints and the herbal ingredients of AHP,^[19,28,29]^ or the effect of and satisfaction with AHP.^[23,30–32]^ However, there is no critical appraisal of the current evidence for the treatment of bronchitis and no systematic review.^[22]^

This systematic review aims to summarize and critically evaluate the evidence from clinical trials that have tested the efficacy of AHP as a treatment for bronchitis.

## 2 Methods

The protocol for this systematic review was prepared according to the Preferred Reporting Items for Systematic Review and Meta-analysis Protocols (PRISMA-P) guidelines.^[33]^

### 2.1 Inclusion criteria

#### 2.1.1 Types of studies

We will include randomized controlled trials (RCTs) or quasi-RCTs comparing AHP with conventional medicine. Case studies, case series, case-control studies, and reviews will be excluded. There will be no restrictions on the year or language of publication.

#### 2.1.2 Types of participants

Male and female participants of any age with clinically diagnosed acute or chronic bronchitis will be included. Studies will be excluded if the participants had other serious medical conditions such as cancer, liver disease, or kidney disease and so on.

#### 2.1.3 Types of intervention and controls

All types of herbal patching will be included, with no limitations in the number, administration method, dosage, the composition of herbal medicine, detail of acupoint, or duration of treatment. Treatment with AHP for the purpose of this review was defined as the use of a herbal preparation patch that covered acupoints for a certain period of time. Comparators will include usual care, no treatment, or western medicine. We will exclude studies using other types of AHP for comparison, or those using a different baseline therapy.

#### 2.1.4 Types of outcome measures

- Primary outcome: Treatment effect (the number of patients whose bronchitis symptoms improved)
- Secondary outcomes:
  1. Quality of life
  2. Adverse events

### 2.2 Search method and strategy

**Figure 1:**
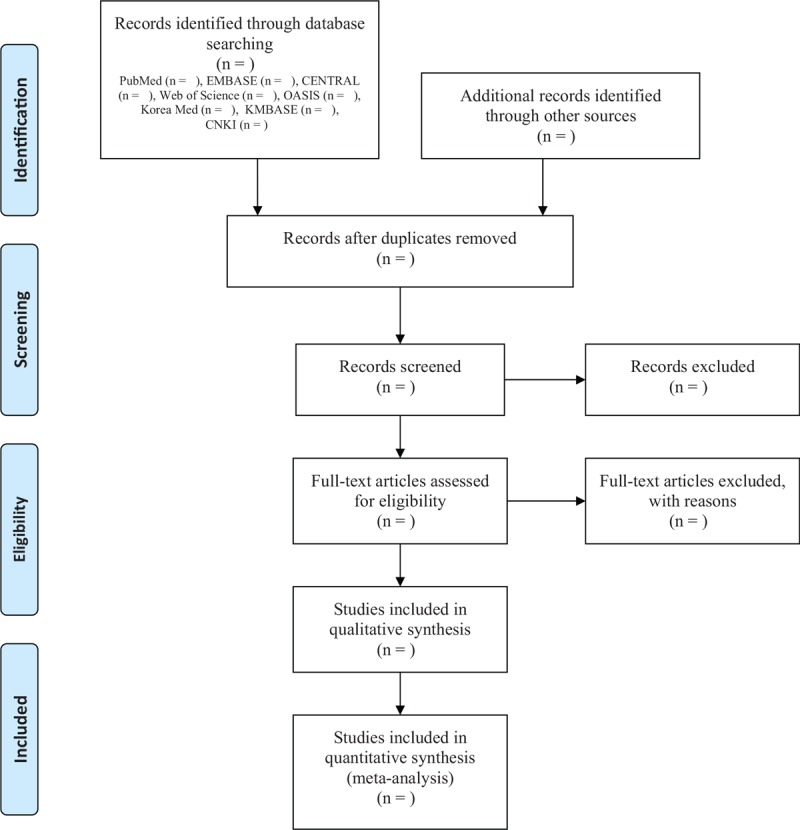
Flow diagram of the randomized controlled trials selection process in this systematic review. The image by Moher et al is available under the CCBY-NC license in the article “Preferred reporting items for systematic reviews and meta-analyses: the PRISMA Statement.”

The databases searched will include PubMed, EMBASE, CENTRAL, Web of Science, 3 major Korean medical databases (OASIS, KoreaMed, and KMBASE) and the Chinese database China National Knowledge Infrastructure (CNKI). We will also search Google Scholar. We will use the following search terms: “acupoint herbal patching” AND “clinical trial” AND “bronchitis.” Searches will be conducted in Korean, English, and Chinese. The process of study selection is presented in the adapted PRISMA flow diagram in Figure 1.

### 2.3 Data extraction and assessment of the risk of bias

#### 2.3.1 Data extraction

Two authors (JHJ and ES) will independently screen the titles and abstracts of the studies identified. Two authors (JHJ and KHK) will subsequently screen the full-text articles of studies to be potentially included, and then perform data extraction and quality assessment using a predefined data extraction form. In the event of disagreement between these 2 authors that they cannot resolve through discussion, an arbiter (SJP) will be consulted, who will make the final decision.

#### 2.3.2 Assessment of risk of bias

Three authors (JHJ, KHK, and LH) will assess the quality of the methodology of each individual study using the Cochrane Collaboration's Risk of Bias Assessment tool (version 5.3).^[34]^ This tool comprises 7 domains: random sequence generation, allocation concealment, blinding of the participants and personnel, blinding of the outcome assessment, incomplete outcome data, selective reporting, and other sources of bias. The results of the assessment will be assigned to either of the 3 categories: low, high, or unclear risk of bias.

### 2.4 Data synthesis

All statistical analyses will be conducted using the Review Manager software (RevMan 5.3). Dichotomous data will be presented as the risk ratio with a 95% confidence interval (CI); continuous data will be reported as mean difference with 95% CI. We will use the Grading of Recommendations Assessment, Development and Evaluation (GRADE) software to determine the quality of evidence based on the Cochrane Handbook for Systematic Reviews of Interventions to create a summary of findings table.^[35]^

#### 2.4.1 Assessment of heterogeneity

Heterogeneity among included studies will be identified using the *I*^2^ test. We will use the random effect model for meta-analysis. If a meta-analysis is possible, we will use the *I*^2^ statistic to quantify inconsistencies across the included studies. A cut-off point of 50% will be deemed to indicate substantial heterogeneity. Where heterogeneity exists, we will conduct a subgroup analysis.

#### 2.4.2 Subgroup analysis and the investigation of heterogeneity

Where studies and data permit, subgroup analyses will be conducted based on the method of AHP, the composition of herbal medicine applied with the patches, age, duration of treatment, or the type of control intervention.

#### 2.4.3 Assessment of reporting biases

If 10 or more studies are included, we will assess funnel plot asymmetry for publication bias. We will assess small study effects using Egger method.

## 3 Discussion

Respiratory diseases caused by air pollution are becoming more severe worldwide. The aim of this study was to provide an update on the treatment effect of AHP by acquiring the literature on its clinical studies. In this review, we will search as comprehensive data sources as possible without any language restrictions. Furthermore, this study aims to provide additional information that will facilitate health policies for the prevention and treatment of bronchitis.

## Author contributions

**Methodology:** Kyeong Han Kim, Eunhye Song, Lin Ang.

**Project administration:** Kyeong Han Kim.

**Writing – original draft:** Ji Hee Jun.

**Writing – review & editing:** Sunju Park.
